# Autophagy Is a Defense Mechanism Inhibiting Invasion and Inflammation During High-Virulent *Haemophilus parasuis* Infection in PK-15 Cells

**DOI:** 10.3389/fcimb.2019.00093

**Published:** 2019-04-16

**Authors:** Chaoxiong Yue, Jinquan Li, Hui Jin, Kexin Hua, Wei Zhou, Yueyi Wang, Guirong Cheng, Dan Liu, Lang Xu, Yushan Chen, Yan Zeng

**Affiliations:** ^1^Brain and Cognition Research Institute, Wuhan University of Science and Technology, Wuhan, China; ^2^Hubei Province Key Laboratory of Occupational Hazard Identification and Control, Wuhan University of Science and Technology, Wuhan, China; ^3^State Key Laboratory of Agricultural Microbiology, College of Veterinary Medicine, Huazhong Agricultural University, Wuhan, China; ^4^Big Data Science and Engineering Research Institute, Wuhan University of Science and Technology, Wuhan, China

**Keywords:** *Haemophilus parasuis*, SH0165, autophagy, invasion, inflammation

## Abstract

Bacterial infections activate autophagy and autophagy restricts pathogens such as *Haemophilus parasuis* through specific mechanisms. Autophagy is associated with the pathogenesis of *H. parasuis*. However, the mechanisms have not been clarified. Here, we monitored autophagy processes using confocal microscopy, western blot, and transmission electron microscopy (TEM) and found that *H. parasuis* SH0165 (high-virulent strain) but not HN0001 (non-virulent strain) infection enhanced autophagy flux. The AMPK/mTOR autophagy pathway was required for autophagy initiation and ATG5, Beclin-1, ATG7, and ATG16L1 emerged as important components in the generation of the autophagosome during *H. parasuis* infection. Moreover, autophagy induced by *H. parasuis* SH0165 turned to fight against invaded bacteria and inhibit inflammation. Then we further demonstrated that autophagy blocked the production of the cytokines IL-8, CCL4, and CCL5 induced by SH0165 infection through the inhibition of NF-κB, p38, and JNK MAPK signaling pathway. Therefore, our findings suggest that autophagy may act as a cellular defense mechanism in response to *H. parasuis* and provide a new way that autophagy protects the host against *H. parasuis* infection.

## Introduction

Autophagy is a double edge sword in microbial infection (Shintani and Klionsky, 2004). Some viruses are eliminated by autophagy, while others use autophagy to benefit their replication, spread, and survival (Richards and Jackson, 2012; Ding et al., 2014). On the bacterial side, autophagy targets bacteria while bacteria inhibit autophagy or promote survival (Fabri et al., 2011; Huang and Brumell, 2014). Specifically, autophagy can target intracellular bacteria Group A *Streptococcus* (GAS), *Mycobacterium tuberculosis*, and *Salmonella enterica* subsp. *enterica* serovar *Typhimurium*, then deliver them into the lysosome for degradation (Nakagawa et al., 2004; Jia et al., 2009; Watson et al., 2012). Although some targeted bacteria are eliminated by autophagy, others have developed diverse strategies to escape the defense system period. For example, *Francisella tularensis, Yersinia enterocolitica*, and *Orientia tsutsugamushi* (Deuretzbacher et al., 2009; Chong et al., 2012; Choi et al., 2013). Moreover, some bacteria exploit autophagy for their growth, for example, *Staphylococcus aureus*, uropathogenic *Escherichia coli* and *Listeria monocytogenes* (Amer et al., 2005; Schnaith et al., 2007; Wang et al., 2012).

*Haemophilus parasuis (H. parasuis)* is the etiological agent of Glässer's disease in swine, which is characterized by a severe systemic inflammation of polyserositis, arthritis and meningitis (Oliveira and Pijoan, 2004; Aragon et al., 2012). It comprises 15 known serotypes to date (Kielstein and Rapp-Gabrielson, 1992). Serovars 1, 5, 10, 12, 13, and 14 are highly virulent and cause high mortality in swine; serovars 2, 4, 8, and 15 show moderate virulence; serovars 3, 6, 7, 9, and 11 are non-virulent (Kielstein and Rapp-Gabrielson, 1992; Howell et al., 2015). Among them, serovars 5 are among the most epidemic isolates and lead to high mortality and morbidity in swine, causing enormous economic losses in the global pig industry (Cai et al., 2005). Our recent progress in SH0165 strains (sevovar 5, highly virulent) have elucidated the mechanisms of inflammation mediated by several inflammatory signaling cascades (Chen et al., 2012, 2015a,b; Ma et al., 2018), and recent evidence showed autophagy is associated with the presence of alive bacteria *H. parasuis* standard strain in cells (Zhang et al., 2016), however, the mechanisms of autophagy involved in cellular invasion and inflammation during *H. parasuis* SH0165 infection are still unknown.

Crosstalk between autophagy and inflammation can balance host defense and homeostasis (Cadwell, 2016). Inflammatory signaling cascades that induce autophagy are subject to be regulated by autophagy, while autophagy can increase or decrease different parts of inflammatory signaling cascades in a context-dependent manner (Cadwell, 2016). It has been reported that increasing autophagy improves survival rate in a mouse model of sepsis and mice with *S. aureus*-mediated bacteremia and pneumonia through the reduction of inflammation (Figueiredo et al., 2013; Cadwell, 2016). Autophagy also can affect the production of inflammatory cytokines in various aspects. Autophagy inhibits the production of cytokines by autophagic degradation of inflammasome components (Harris et al., 2011; Shi et al., 2012) or blocking the inflammatory signaling pathway (Cadwell, 2016; Li et al., 2017; Zhang et al., 2017). Conversely, loss of autophagy in macrophages or dendritic cells, either by treatment with 3-Methyladenine, or through knockdown of autophagy-related protein (ATG), stimulates the secretion of IL-1β (Crişan et al., 2011). Thus, how the function of autophagy is integrated into inflammatory signaling cascades induced by *H. parasuis* infection warrants further investigation.

In this study, we sought to investigate whether SH0165 (serovar 5, high-virulent strain) and HN0001 (serovar 6, non-virulent strain) infection induces autophagy and the specific role of autophagy in bacterial invasion and inflammation during *H. parasuis* infection. Moreover, we explored the mechanism underlying autophagy regulated inflammation through inflammatory signaling cascades during *H. parasuis* infection. This observation could provide useful information for further understanding the role of autophagy in *H. parasuis* infection and improve our knowledge of new strategies against this pathogen.

## Materials and Methods

### Bacterial and Cells Culture

Clinical isolated strains of *H. parasuis* SH0165 (serovars 5, high-virulent clinical strain) and HN0001(serovars 6, non-virulent clinical strain) (Cai et al., 2005) were cultured in tryptic soy broth (TSB; Difco Laboratories, Detroit, MI, USA) supplemented with 5% bovine serum and 0.2 mg/ml nicotinamide adenine dinucleotide (NAD) at 37°C overnight. Porcine kidney (PK-15) cells were maintained at 37°C and 5% CO_2_ in Dulbecco's Modified Eagle Media (DMEM, Gibco), supplemented with 10% fetal bovine serum (FBS, Gibco), 100 U/mL penicillin, and 10μg/mL streptomycin.

### Reagents, Antibodies, and siRNA

Rapamycin (Rapa), 3-methyladenine (3MA), chloroquine (CQ), and Bafilomycin A1 (Baf-A1) were purchased from MedChem Express (Monmouth Junction, NJ, USA). Cells were pretreated with 1 μM rapamycin, 3 μM 3 MA, 10 μM CQ or 10 nM Baf-A1 before infection with *H. parasuis*. Recombinant adenovirus tandom expressing GFP-RFP-LC3 was obtained from HANBIO (Shanghai, China). Primary antibodies used in this study were: rabbit anti-LC3A/B polyclonal IgG (12741), NF-κB p65 (6956), phospho-NF-κB p65 (Ser536) mAb (3031), p38 MAPK (8690), phospho-p38 MAPK (Thr180/Tyr182) rabbit mAb (4511), JNK2 rabbit mAb (9258), phospho-SAPK/JNK (Thr183/Tyr185) rabbit mAb (4668), AMPKα rabbit mAb (5831), phospho-AMPKα (Thr172) rabbit mAb (2535), mammalian target of rapamycin (mTOR) rabbit mAb (2983), and phospho-mTOR (Ser2448) rabbit mAb (5536) were purchased from Cell Signaling Technology (CST, Boston, MA, USA). Rabbit anti-ATG5 polyclonal IgG, rabbit anti-Beclin-1 polyclonal IgG, rabbit anti-ATG7 polyclonal IgG, rabbit anti-ATG16L1 polyclonal IgG, and rabbit monoclonal antibody against β-actin were purchased from ABclonal Biotech (Cambridge, MA, USA). The siRNA targeting ATG5, Beclin-1, ATG7 and ATG16L1 mRNA were synthesized by GenePharma (China) and the sequences are listed in Table 1. Pharmacological treatments and RNA interference do not affect cell viability (Figure S1).

**Table 1:** The sequences of siRNAs used in this study.

**siRNA**	**Sequences (5^**′**^–3^**′**^)**
siATG5	5′-GGAUGUAAUUGAAGCUCAUTT-3′
	5′-AUGAGCUUCAAUUACAUCCTT-3′
siBeclin-1	5′-CCUGGAUCGUGUUACCAUUTT-3′
	5′-AAUGGUAACACGAUCCAGGTT-3′
siATG7	5′-GCAGCUCAUCGAAAGCCAUTT-3′
	5′-AUGGCUUUCGAUGAGCUGCTT-3′
siATG16L1	5′-GGAGGUGUUUGGAGACAAATT-3′
	5′-UUUGUCUCCAAACACCUCCTT-3′
siNegative Control	5′-UUCUCCGAACGUGUCACGUTT-3′
	5′-ACGUGACACGUUCGGAGAATT-3′

### Transfection, Luciferase Reporter Assay, and Infection

Lipofectamine RNAiMAX and Lipofectamine 3000 transfection reagent were purchased from Invitrogen (Carlsbad, CA, USA). PK-15 cells were seeded in 24-well plates the day before transfection at 60–70% confluency. Cells were transfected with 40 pmol/well negative control siRNA or other siRNA by Lipofectamine RNAiMAX transfection reagent. Twenty-four hours after transfection, cells were infected with *H. parasuis* and then harvested for further analyses at the indicated time points. In selected experiments, cells were co-transfected with 100 ng/well of NF-κB luciferase reporter plasmid (pNF-κB-Luc) and 100 ng/well of the Renilla luciferase construct pRL-TK (Promega) together with 40 pmol/well siRNA. Twenty-four hours later, cells were infected or uninfected with *H. parasuis*. Cells were harvested at the indicated time point and luciferase activity was measured using a dual-luciferase Assay System (Promega) according to the manufacturer's directions. Data represent relative firefly luciferase activity normalized to Renilla luciferase activity. Until optical density of *H. parasuis* become 0.8, bacteria were harvested by centrifugation at 5,000 g for 10 min, washed 3 times with PBS and resuspended in DMEM for the infection experiment.

### RNA Isolation and Quantitative Real-Time Polymerase Chain Reaction (qRT-PCR)

Total RNA was collected from PK-15 cells by using TRIzol reagent (Invitrogen, Carlsbad, CA, USA) according to the manufacturer's protocol. RNA was reverse-transcribed into cDNA using iScriptTM cDNA Synthesis Kit (Bio-Rad, Hercules, California, USA), and cDNA amplification was performed by iTaqTM Universal SYBR Green supermix (Bio-Rad, Hercules, California, USA). Each sample has triplicate duplication measurements and porcine glyceraldehyde-3-phosphate dehydrogenase (GAPDH) was used as endogenous control. Forward/reverse primers used for qRT-PCR are shown in Table 2.

**Table 2:** The sequences of primers used in this study.

**Primer**	**Sequences (5^**′**^–3^**′**^)**
ATG5-F	5′-AACACTGCTGCAAGCCTAAC-3′
ATG5-R	5′-CATGTCGCAGCTGAAGTTGA-3′
Beclin-1-F	5′-TGTCACCATCCAGGAACTCA-3′
Beclin-1-R	5′-CTGTTGGCACTTTCTGTGGA-3′
ATG7-F	5′-TGTGAGTCGTCCAGGATTGG-3′
ATG7-R	5′-GCAAAACAGATACCATCAATTCCA-3′
ATG16L1-F	5′-TCTGGGAGGTGTTTGGAGAC-3′
ATG16L1-R	5′-CACAGTCCAGATTCGGCTTG-3′
CCL4-F	5′-AGCGCTCTCAGCACCAATG-3′
CCL4-R	5′-TCCGCACGGTGTATGTGAA-3′
CCL5-F	5′-CAGCATCAGCCTCCCCATA-3′
CCL5-R	5′-GGGCGGGAGAGGTAGGAAA-3′
IL-8-F	5′-AGTTTTCCTGCTTTCTGCAGCT-3′
IL-8-R	5′-TGGCATCGAAGTTCTGCACT-3′
GAPDH-F	5′-CCCCAACGTGTCGGTTGT-3′
GAPDH-R	5′-CCTGCTTCACCACCTTCTTGA-3′

### Western Blot

Protein samples from cells were collected and homogenized in RIPA buffer (thermo scientific, 81 Wyman Street, Waltham, MA) containing the protease and phosphatase inhibitors cocktail (Sigma-Aldrich, St. Louis, MO, USA). After separating by SDS-PAGE electrophoresis using 10% acrylamide gel, proteins were transferred to a PVDF membrane. After incubation with primary and secondary antibodies, immunoreactive bands were visualized by Clarity™ Western ECL Substrate (Bio-Rad, Hercules, California, USA). The intensities of individual bands were quantified with densitometry using ImageJ 1.48 software (National Institutes of Health, Bethesda, MD, USA). All blots were reprobed with β-actin antibody and normalized to the β-actin level.

### Confocal Laser Scanning Microscopy

PK-15 cells were grown on microscopy cover glasses in 24 well plates and infected with adenovirus expressing GFP-RFP-LC3 at 1 × 10^8^ TU/ml (MOI = 10). Twenty-four hours after adenovirus transduction, cells were treated with rapamycin, 3-MA, CQ or infected with *H. parasuis* for indicated times. Cells were washed with PBS, fixed with 4% paraformaldehyde, mounted with 4,6-diamidino-2-phenylindole (DAPI, Beyontime, China) nucleic acid stain. Fluorescence of green fluorescent protein-LC3 (GFP-LC3) and red fluorescent protein-LC3 (RFP-LC3) were observed under FV1000 laser scanning confocal microscope (FluoView1000, Olympus, Tokyo, Japan). The average number of GFP-LC3 and RFP-LC3 puncta per cell from at least 30 cells per sample was counted (Mizushima et al., 2010; Ni et al., 2011).

### Transmission Electron Microscopy (TEM)

TEM was employed to identify autophagosomes as previously described (Owen et al., 2014; Gu et al., 2018). PK-15 cells infected or uninfected with *H. parasuis* were fixed in 2.5% glutaraldehyde. After washed with PBS, cells were postfixed in 1% osmium tetroxide in 0.1 M sodium cacodylate, followed by dehydration in a graded series of ethyl alcohol washes before being embedded. Ultrathin sections (80 nm) were cut using a Leica ultracut ultramicrotome (EM UC7, Leica, Germany) and viewed on an electron microscope (Tecnai G2 20 TWIN, FEI, USA). For each experimental group, the autophagosomes of 15 cellular cross-sections were counted.

### Cell Invasion Assays

The invasion assay was performed as previously reported (Vanier et al., 2006) with slight modifications. PK-15 cells were grown until confluent monolayers in 24-well plates and infected with indicated dose of *H. parasuis*. The plates were centrifuged at 500 × g for 5 min to enhance the contact of bacteria with the surface of the monolayer. The plates were incubated for 3 h at 37°C with 5% of CO_2_ to allow bacteria to invade cells. Cells were then vigorously washed five times with PBS to remove non-specific bacterial attachment and culture medium containing two antibiotics (100 mg/mL penicillin and 25 μg/mL gentamicin, Sigma) was added. The plates were incubated for 1 h at 37°C in 5% of CO_2_ to kill extracellular bacteria. After washing three times, cells were incubated with 0.025% trypsin for 20 min at 37°C. After the incubation, cells were vigorously scraped with a sterile cell scraper. The new cell suspensions were diluted and put onto TSA plates and incubated for 48 h at 37°C. The invaded bacteria were calculated as the average number per well. All the assays were performed in triplicate.

### MTT

Cell viability was determined by the MTT [3-(4,5-dimethyl-2-thiazolyl)-2,5-diphenyl-2-H-tetrazolium bromide] method according to the manufacturer's instructions (Sigma-Aldrich, St. Louis, MO, USA). Briefly, cells were cultured in 96-well plate for 24 h. Then some wells were replaced by culture media containing DMSO, rapamycin, 3-MA or CQ and other wells were transfected with siRNAs. Following treatment, cells were added with 10 μl of MTT (5 mg/ml) for 4 h at 37°C. After removing the supernatants, the culture medium was replaced with 150 μl DMSO to dissolve the precipitate. Absorbance was measured at 570 nm using the SpectraMax M2 spectrophotometer (Molecular Devices, Sunnyvale, CA, USA). All tests were performed in triplicates. The viability of cells was expressed as a percentage of that of untreated control cells.

### Statistical Analysis

The results were shown as mean ± standard deviation (SD) from at least three independent assays. Data were analyzed for statistical significance using one-way analysis of variance (ANOVA). A *P*-value<0.05 was considered significant and a *P*-value<0.01 was considered highly significant.

## Results

### *H. parasuis* SH0165 Infection Induces LC3 Punctation and LC3-II Formation

**Figure 1:**
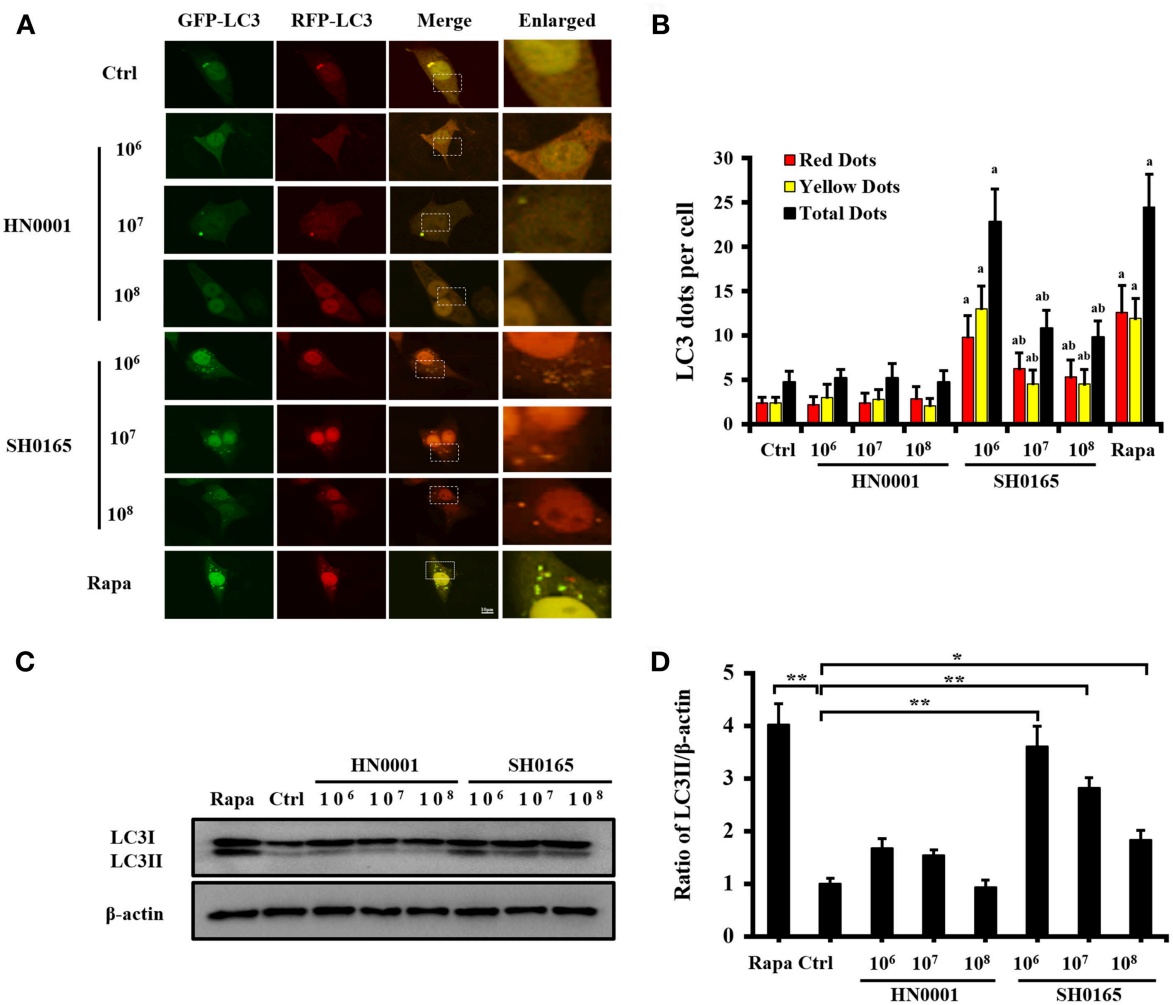
High-virulent *H. parasuis* SH0165 infection induced LC3 punctation in PK-15 cells. PK-15 cells were infected with *H. parasuis* (SH0165 or HN0001) at 10^6^, 10^7^, or 10^8^ CFU/mL for 6 h or uninfected (Ctrl). Before infection, cells were treated with rapamycin (Rapa) (1 μM, 12 h) as positive control. **(A)** PK-15 cells were infected with adenovirus expressing GFP-RFP-LC3 for 24 h followed by treatment with SH0165, HN0001, or rapamycin. Representative images of fluorescent LC3 puncta are shown. Scale bars, 10 μm. **(B)** Average number of yellow LC3 dots and red LC3 dots per cell in each condition was quantified. Total LC3 dots are the addition of the number of yellow LC3 dots with red LC3 dots. More than 30 cells were counted in each condition and data (mean ± SD) represent three independent experiments. ^a^*P* < 0.05 compared with the control group; ^b^*P* < 0.05 compared with the SH0165 (10^6^ CFU/mL) group. **(C,D)** SH0165- and HN0001- infected cell lysates were analyzed for LC3 protein by Western blot. Densitometry was applied to quantify LC3 and β-actin protein density. The ratio of normalized LC3-II to β-actin; the data were presented as a mean ± SD from three independent experiments. *P < 0.05 vs. control group; **P < 0.01 vs. control group.

To investigate whether SH0165 and HN0001 infection induce autophagy, PK-15 cells were infected with adenovirus GFP-RFP-LC3, followed by infection with SH0165 and HN0001 in a time- and dose-dependent manner. GFP is sensitive to acid, while RFP is resistant to both acid and lysosomal proteases (Hariharan et al., 2011). Green LC3 puncta primarily indicate autophagosomes, whereas red LC3 puncta indicate both autophagosomes and autolysosomes. Yellow puncta, overlay of red and green fluorescent, are indicators of autophagosomes, while the free red puncta represent autolysosomes (Kimura et al., 2007; Hariharan et al., 2011). As shown in Figure 1A, autophagosomes and autolysosomes were typically observed in SH0165 and rapamycin (autophagy inducer) treated cells, while few autophagic features were observed in control and HN0001 infected cells (Figure 1A). SH0165 infection with 10^6^ CFU/mL in PK-15 cells induced the highest LC3 punctation and relative proportion of autophagosomes to autolysosomes, indicating the maturation of autophagosomes (Figures 1A,B).

The transformation of LC3 (LC3-I) to its autophagosomal-associating form (LC3-II), indicates autophagosome formation (Mizushima et al., 2010). Our results showed that SH0165 infection with 10^6^, 10^7^, and 10^8^ CFU/mL led to a significant increase in LC3-I to LC3-II conversion, while HN0001 failed to induce LC3-II formation (Figures 1C,D). 10^7^ and 10^8^ CFU/mL SH0165 induced smaller amounts of LC3-II, which may be due to the increased cell death with higher infection doses. Some pathogens induced autophagy at the early stages of infection but not at the late stages (Birmingham et al., 2006; Fan et al., 2017). We detected that SH0165 infection with 10^6^ CFU/mL induced LC3-II formation at 3 and 6 h post-infection, as compared to the uninfected control cells (Figures S2A,B). However, at 9, 12, and 24 h post-infection, LC3-II gradually decreased due to the increased cell death with longer infection time (Figures S2A,B). BafA1 is an inhibitor often used to inhibit autophagic flux at the last stage (Klionsky et al., 2016). Figure S3 showed that BafA1+SH0165 group enhanced the amount of LC3-II compared with SH0165 group at 10^6^ and 10^7^ CFU/mL, suggesting the autophagy activation induced by SH0165.

### *H. parasuis* SH0165 Infection Induces Autophagosome Formation

**Figure 2:**
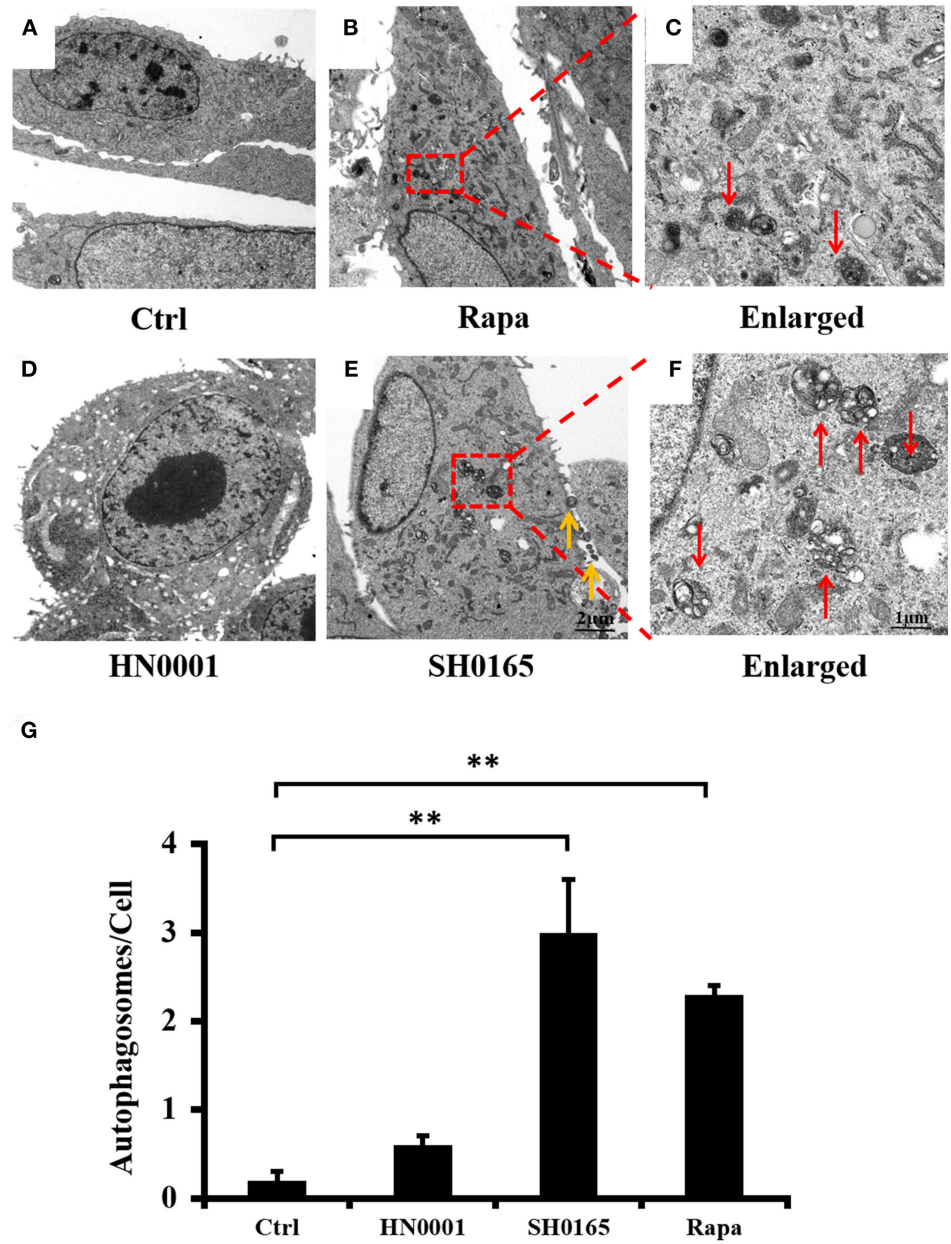
*H. parasuis* SH0165 infection induced autophagosome accumulation in PK-15 cells. Ultrastructural analysis of *H. parasuis*-induced autophagy by transmission electron microscopy (TEM) in PK-15 cells uninfected **(A)** or infected with SH0165 **(E)** or HN0001**(D)** (10^6^ CFU/mL) for 6 h (500 magnification) or treated with Rapa **(B)**. The right panels **(C,F)** showed the magnified image of the area indicated by the box in the left panels. The red arrows indicate autophagosomes. The yellow arrows indicate the bacteria. Scale bars, 1 or 2 μm. **(G)** The number of autophagosome per cell in each TEM section (*n* = 20 cells). Data were shown as mean ± SD (**P < 0.01 vs. control group). Ctrl: uninfected as control. Rapa: rapamycin.

Transmission electron microscopy (TEM) is the valid standard technique to identify autophagosomes at a resolution in the nm range (Mizushima et al., 2010). To further determine whether *H. parasuis* infection could induce autophagosome formation, TEM is used to observe the ultrastructure of autophagy in PK-15 cells. As shown in Figure 2, an accumulation of autophagosomes was observed in SH0165- and rapamycin-treated cells (Figures 2B,E,G), as compared to the uninfected control and HN0001-infected cells (Figures 2A,D,G). Figures 2C,F showed the higher magnification of the autophagosomes in rapamycin- and SH0165-treated cells. Thus, the morphological results obtained by TEM confirm that autophagosome formation is consistent with the accumulation of LC3 punctation.

### 3-Methyladenine Inhibits Autophagy Induced by *H. parasuis* SH0165

**Figure 3:**
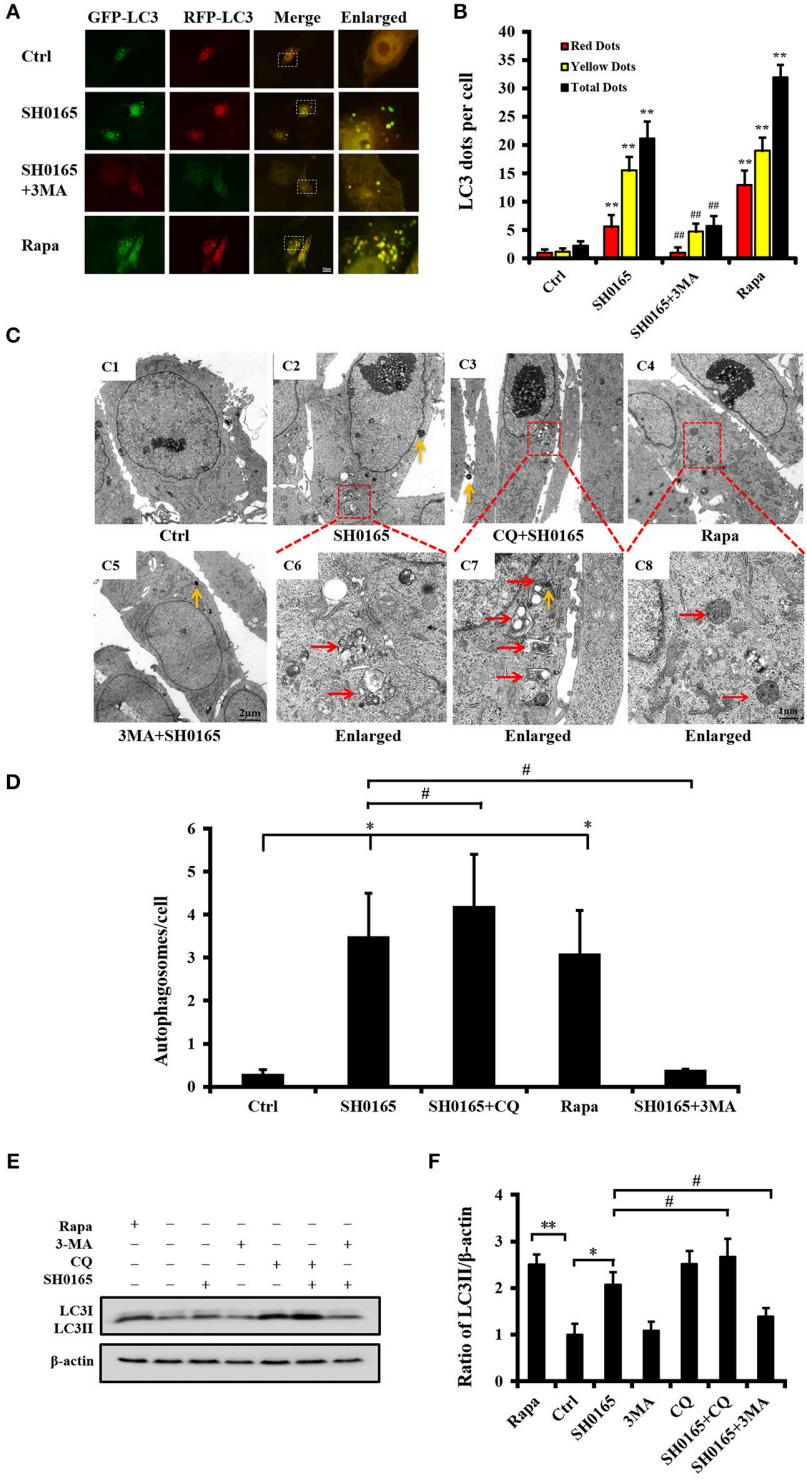
**3**-Methyladenine (3MA) inhibit autophagy induced by *H. parasuis* SH0165. PK-15 cells were infected with *H. parasuis* SH0165 or uninfected (Ctrl) at 10^6^CFU/mL for 6 h. Before infection, cells were treated with rapamycin (Rapa: 1 μM, 12 h), 3-MA (3 mM, 3 h) or chloroquine (CQ: 10 μM, 3 h). **(A)** PK-15 cells were infected with adenovirus expressing GFP-RFP-LC3 for 24 h followed by treatment with SH0165, Rapa, or 3-MA. Autophagosomes were observed by confocal microscopy. Scale bar, 10 μm. **(B)** Average number of puncta in each cell from at least 30 cells in each treatment. Data are reported as mean ± SD of three independent experiments (*n* = 3; **P < 0.01 vs. control group and ^##^*P* < 0.01 vs. SH0165 group). **(C)** Autophagic vacuoles in cells observed by TEM. **(C1)** showed control group and **(C5)** showed 3MA plus SH0165 infection. The below panels **(C6)**–**(C8)** showed the magnified image of the area indicated by the box in the above panels **(C2)**–**(C4)**. The vesicles with characteristics of autophagosomes are indicated by red arrows. The yellow arrows indicate the bacteria. Scale bar: 1 or 2 μm. **(D)** The number of autophagosome per cell in each TEM section (*n* = 20 cells). Data were shown as mean ± SD (*P < 0.05 vs. control group, ^#^*P* < 0.05 vs. SH0165 group). **(E,F)** Analysis of LC3-II expression and its ratio to β-actin that was normalized to un-infection (Ctrl) set at 1.0. Data are reported as mean ± SD (*n* = 3; *P < 0.05, **P < 0.01 vs. control group and ^#^*P* < 0.05 vs. SH0165 group).

To further identify the role of autophagy induced by SH0165 in PK-15 cells, an autophagy-specific inhibitor, 3-MA, was added into the cells. Compared with the SH0165 group, the SH0165+3-MA group showed significant decreased LC3 punctation, which indicates that 3-MA inhibited the autophagy induced by SH0165 (Figures 3A,B). Subsequently, we analyzed the formation of autophagosome using TEM (Figures 3C,D), and found consistent results with LC3 punctation assays. As shown in Figures 3E,F, the ratio of LC3-II to β-actin was significantly lower in SH0165+3-MA groups than those in SH0165 group. Taken together, 3-MA had the effects on inhibiting SH0165-induced autophagy. To verify the role of autophagosomes fusing with lysosomes in LC3-II degradation in SH0165-infected cells, the turnover of LC3-II after chloroquine (CQ, a commonly used autophagy inhibitor) treatment were analyzed. Under TEM, we detected a large number of autophagosomes with vacuoles of PK-15 cells in CQ+SH0165 groups (Figure 3C). Also as shown in Figures 3E,F, CQ+SH0165 group significantly increased the ratio of LC3-II/β-actin compared with SH0165 group, suggesting that SH0165 infection indeed enhanced the autophagic flux. These results revealed that by blocking the last step of the autophagy pathway, CQ treatment leads to the accumulation of ineffective autophagosomes.

### *H. parasuis* SH0165 Induces Autophagic Pathway

**Figure 4:**
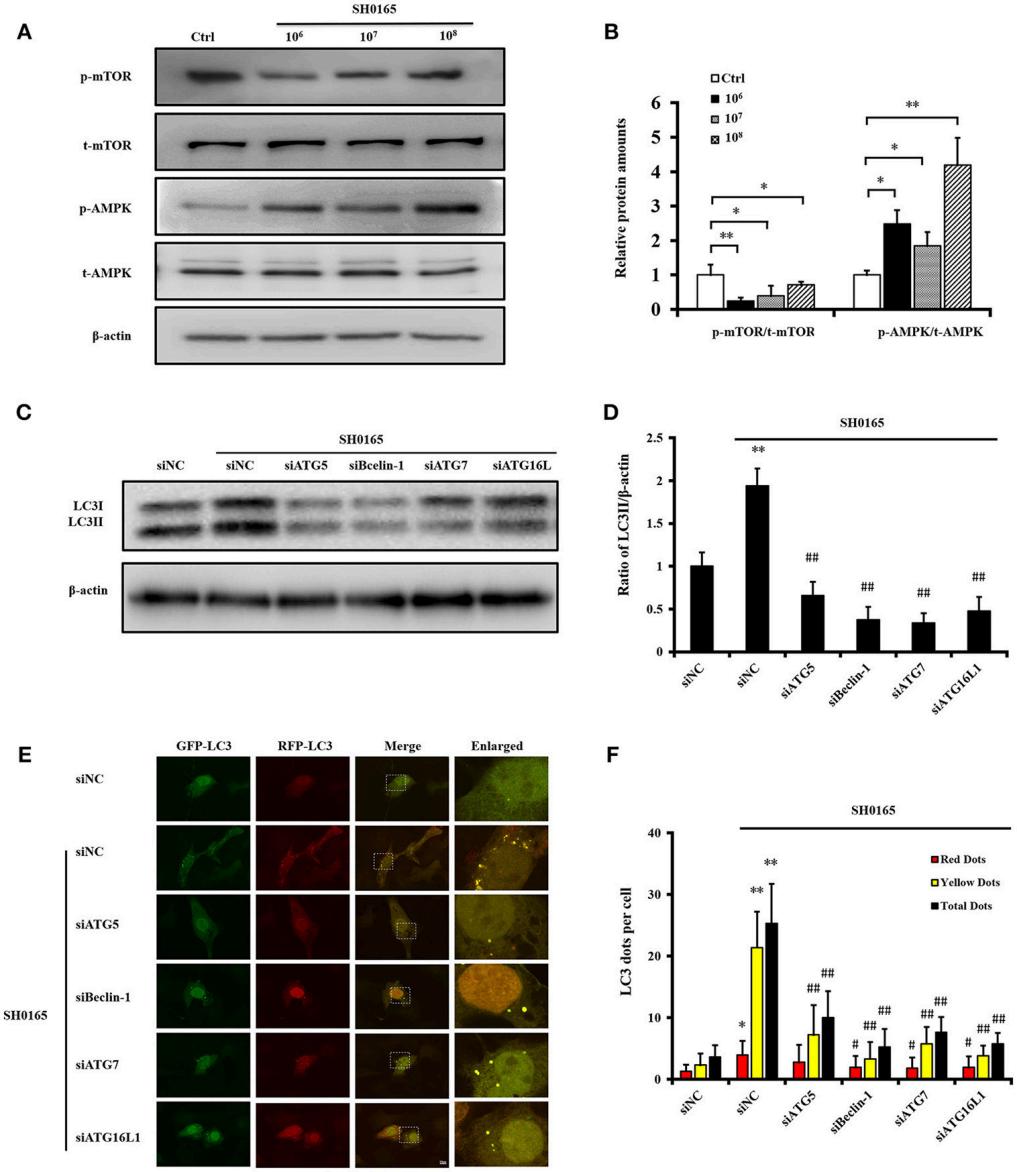
Classical autophagy signaling pathway were involved in autophagy induced by *H. parasuis* SH0165. **(A)** PK-15 cells were infected with SH0165 or uninfected (Control) at 10^6^, 10^7^, or 10^8^ CFU for 6 h. Cell lysates were prepared at 6 h post-infection and analyzed for the expression of p-AMPK, p-mTOR, total AMPK, and mTOR by Western blot. **(B)** Protein density was analyzed by the software ImageJ. Standard deviation represents three independent experiments. *P < 0.05 and *P < 0.01 vs. control group. **(C)** PK-15 cells were transfected with siNegative, siATG5, siBeclin-1, siATG7, or siATG16L1 and un-infected or infected with SH0165 at 10^6^ CFU/mL. Whole cell extracts were subjected to Western blot analysis with antibodies specific for LC3 and β-actin. **(D)** Relative levels of LC3-II in comparison to β-actin in each group have been shown as histogram. Data are presented as means ± SD of three independent experiments. Statistical significance compared with the control group is indicated by **P < 0.01, ^##^*P* < 0.01 vs. siNC plus SH0165 group. **(E)** PK-15 cells were transfected with siNegative, siATG5, siBeclin-1, siATG7, or siATG16L1 and infected with adenovirus expressing GFP-RFP-LC3 for 24 h followed by infected with SH0165. Representative images of fluorescent LC3 puncta are shown. Scale bars, 10 μm. **(F)** Average number of yellow LC3 dots and red LC3 dots per cell in each condition was quantified. Total LC3 dots are the addition of the number of yellow LC3 dots with red LC3 dots. More than 30 cells were counted in each condition and data (mean ± SD) represent three independent experiments. *P < 0.05, **P < 0.01 vs. siNC group; ^#^*P* < 0.05, ^##^*P* < 0.01 vs. siNC plus SH0165 group.

Previous reports have demonstrated that AMP-activated protein kinase (AMPK) and mammalian target of rapamycin (mTOR) coordinated autophagy initiation in mammalian cells (Yang et al., 2014). In our analysis of the AMPK/mTOR autophagy pathway induced by SH0165, we found that the phosphorylation levels of AMPK were obviously increased while the phosphorylations of mTOR were declined in a dose-dependent manner in SH0165 infected groups compared the control group (Figures 4A,B). These results suggested that SH0165 activated the AMPK/mTOR autophagy pathway. The generation of the autophagosome is mediated by the sequential activities of the key protein complexes which are comprised of autophagy related (ATG) proteins (Cadwell, 2016). Among them, ATG5, Beclin-1, ATG7, and ATG16L1 have been shown to play a critical role in the canonical autophagy pathway (Gomes and Dikic, 2014). To determine whether these proteins contributed to the autophagy induced by SH0165, siRNAs of ATG5, Beclin-1, ATG7, and ATG16L1 effects on LC3 protein were evaluated in PK-15 cells infected with SH0165. Firstly, we confirmed at the RNA and protein levels that ATG5, Beclin-1, ATG7 and ATG16L1 expression were silenced (Figure S4). After transfection with siRNAs of ATG5, Beclin-1, ATG7 and ATG16L1, PK-15 cells were uninfected or infected with SH0165. As expected, ATG5, Beclin-1, ATG7 and ATG16L1 deficiency led to impaired conversion of endogenous LC3-I to LC3-II (Figures 4C,D) and decreased number of LC3 puncta (Figures 4E,F) following SH0165 infection. These results demonstrated that the knockdown of ATG5, Beclin-1, ATG7, and ATG16L1 effectively blocked autophagy induction of SH0165.

### Autophagy Limits Infection of PK-15 Cells by *H. parasuis* SH0165

**Figure 5:**
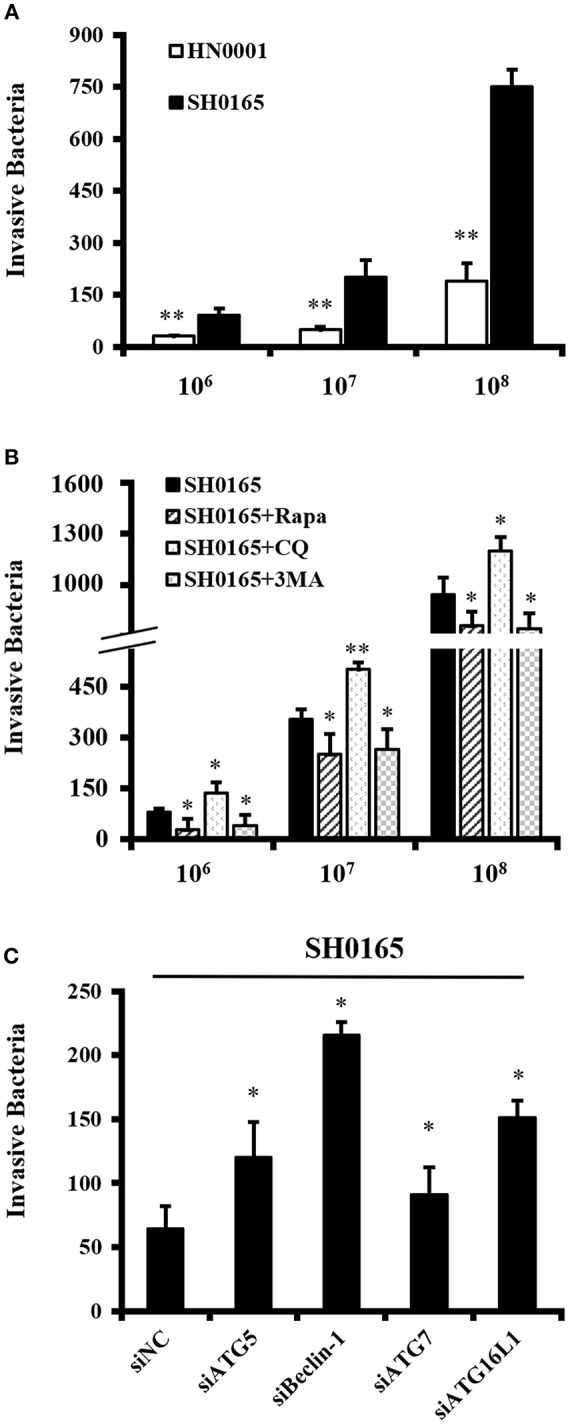
Autophagy inhibits *H. parasuis* SH0165 invasion in PK-15 cells. Invasion assay were performed as described in Materials and methods. The data shown as mean ± SD represent the number of bacteria that invaded the cells from three independent experiments performed in triplicate. **(A)** Invasion of SH0165 and HN0001 at 10^6^, 10^7^, and 10^8^ CFU/mL in PK-15 cells. **P < 0.01 as compared with SH0165 group. **(B)** PK-15 cells were pretreated with rapamycin (Rapa: 1 μM, 12 h), 3-MA (3 mM, 3 h) or chloroquine (CQ: 10 μM, 3 h) followed by SH0165 infection at 10^6^, 10^7^, and 10^8^ CFU/mL. *P < 0.05 and **P < 0.01 as compared with SH0165 group. **(C)** PK-15 cells were transfected with siNegative, siATG5, siBeclin-1, siATG7, or siATG16L1 followed by infected with SH0165 at 10^7^ CFU/mL. *P < 0.05 as compared with siNC plus SH0165 infection group.

Increasing evidence suggests that autophagy combat with various pathogenic bacteria by restricting bacterial invasion and growth (Huang and Brumell, 2014; Escoll et al., 2016; Zhang et al., 2016). In order to explore the role of autophagy in *H. parasuis* SH0165-infecting host cells, the invasion assay was performed. Firstly, the invasion capacities of SH0165 and HN0001 were investigated. As illustrated in Figure 5A, HN0001 had an ~4-fold decline in a dose-dependent manner in invasion compared with SH0165. Then autophagy inducer or inhibitors were used.

PK-15 cells were treated with rapamycin, 3-MA or CQ, and then infected with 10^6^, 10^7^, and 10^8^ CFU/mL of SH0165. As shown in Figure 5B, the invasion assay showed a significant reduction of the number of intracellular bacteria in rapamycin or 3MA plus SH0165 group compared with SH0165 group. Moreover, there was significantly more intracellular bacteria by autophagy inhibitor CQ+SH0165 than SH0165 infection alone. The results in Figure S5 showed the pre-treatment of cells with rapamycin, 3-MA or CQ do not influence the infection rate. Finally, Figure 5C showed knockdown of ATG5, Beclin-1, ATG7, or ATG16L1 in SH0165-infected cells had a statistically significant increase in intracellular load of SH0165. In sum, these results indicate that autophagy inhibited intracellular load of bacteria either by affecting invasion and/or survival of the bacteria.

### Autophagy Serves as a Key Negative Regulator of Inflammation Induced by *H. parasuis* SH0165

**Figure 6:**
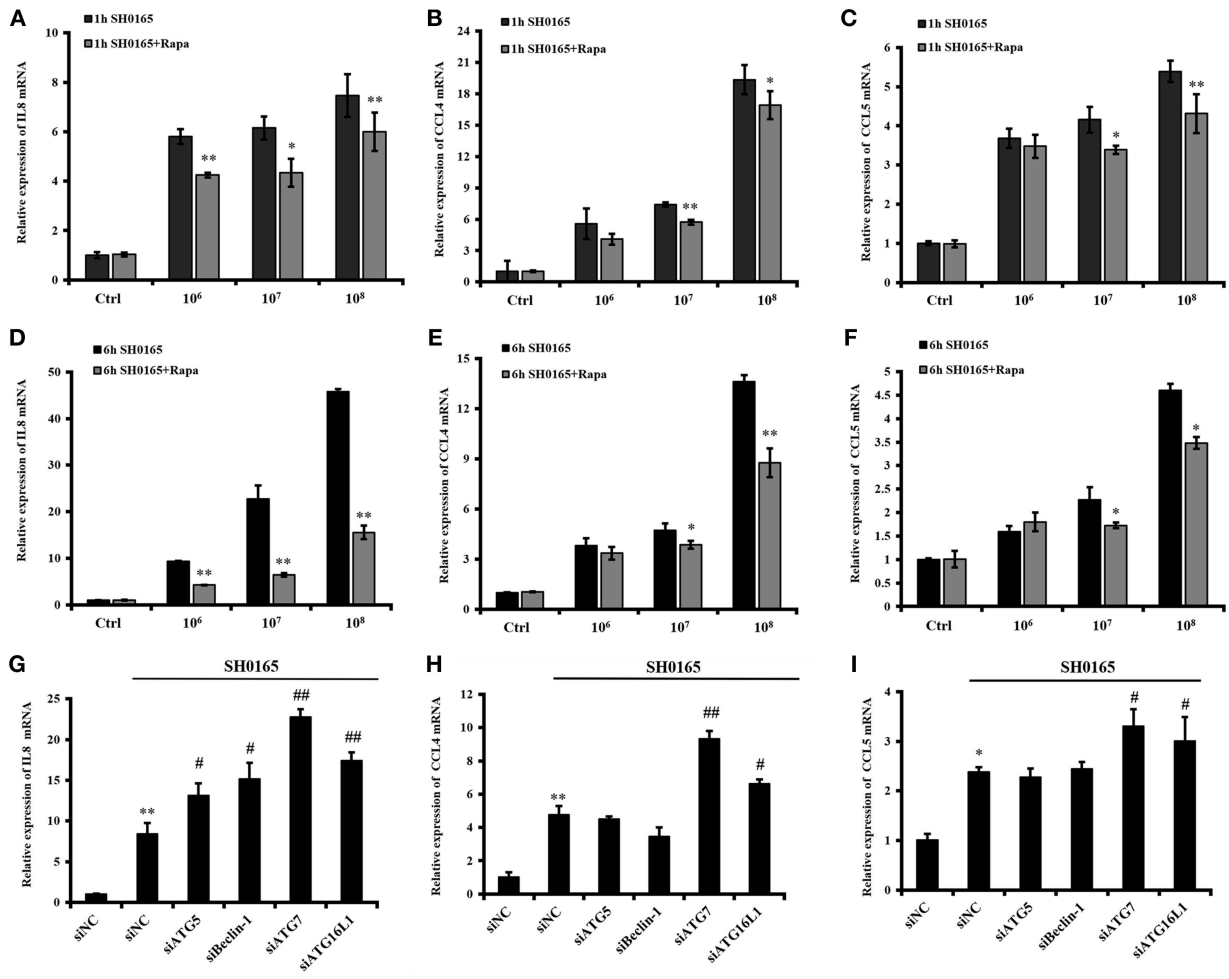
Autophagy inhibits inflammation induced by *H. parasuis* SH0165 in PK-15 cells. **(A)** After treatment with rapamycin for 12 h, PK-15 cells were infected with 10^6^, 10^7^, and 10^8^ CFU/mL of SH0165 for 1 or 6 h, and then cells were harvested and real-time RT-PCR for IL8 **(A,D)**, CCL4 **(B,E)**, and CCL5 **(C,F)** were performed. The data were presented as a mean ± SD from three independent experiments. After transfected with siATG5, siBeclin-1, siATG7, and siATG16L1 for 36 h, PK-15 cells were infected with 10^6^ CFU/mL of SH0165 for 6 h, and then cells were harvested and real-time RT-PCR for IL8 **(G)**, CCL4 **(H)**, and CCL5 **(I)** were performed. The data were presented as a mean ± SD from three independent experiments. *P < 0.05 and **P < 0.01 as compared with SH0165 infection or siNC group. ^#^*P* < 0.05 and ^##^*P* < 0.01 as compared with siNC plus SH0165 infection group.

Recent developments reveal a crucial role for the autophagy pathway in immunity and inflammation (Deretic et al., 2013; Cadwell, 2016; Netea-Maier et al., 2016). After treatment with rapamycin for 12 h, PK-15 cells were infected with 10^6^, 10^7^, and 10^8^ CFU/mL of SH0165 for 1 or 6 h, and then the expressions of IL-8, CCL4, and CCL5 were analyzed. We found that PK-15 cells treated with rapamycin plus 10^6^, 10^7^, and 10^8^ CFU/mL of SH0165 showed a decreased IL-8 and CCL4 compared to SH0165 infection alone (Figures 6A,B,D,E). CCL5 expression were significantly decreased in PK-15 cells treated with rapamycin plus 10^7^, 10^8^ CFU/mL of SH0165 compared with SH0165 infection alone (Figures 6C,F). To further investigate the role of autophagy in SH0165-induced inflammation, cytokines induced by SH0165 were analyzed after the knockdown of ATG5, Beclin-1, ATG7 and ATG16L1. As shown in Figures 6G,H,I, interference of ATG5, Beclin-1, ATG7 and ATG16L1 enhanced the expression of IL8, and interference of ATG7 and ATG16L1 enhanced the expression of CCL4 and CCL5. As a whole, these results show that autophagy suppresses cytokine responses after this bacterial infection.

### Autophagy Inhibits NF-κB, p38/JNK MAPK Signaling Pathway During SH0165 Infection

**Figure 7:**
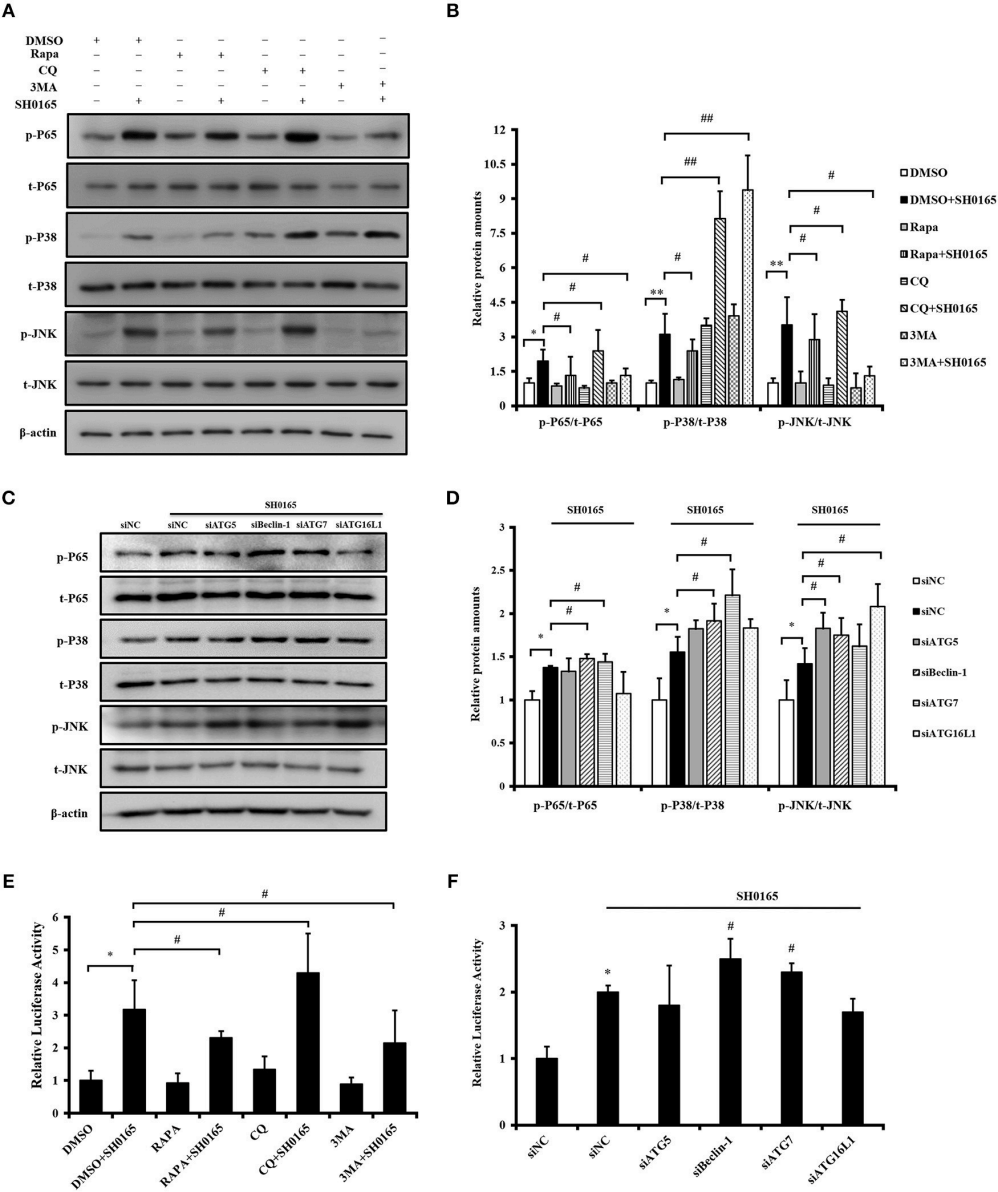
Autophagy inhibits SH0165-induced NF-κB, p38/JNK MAPK signaling pathway. **(A,B,E)** PK-15 cells were treated with rapamycin, 3MA or CQ and then inoculated or un-inoculated with SH0165. **(C,D,F)** PK-15 cells were treated with siRNA of ATG and then inoculated or un-inoculated with SH0165. **(A–D)** Whole cell extracts were prepared at indicated time points and subjected to Western blot analysis with antibodies specific for pp65, pp38, pJNK, and their total protein p65, p38, JNK. pp65/p65, p-p38/p38, and p-JNK/JNK protein levels were quantitated by densitometric analysis using ImageJ software. **(E**,**F)** NF-κB luciferase reporter assay: cells were harvested at 6 h post-infection to measure luciferase activity. Results shown are representative of three independent experiments. *P < 0.05 and **P < 0.01 as compared with DMSO or siNC group. ^#^*P* < 0.05 and ^##^*P* < 0.01 as compared with siNC or DMSO plus SH0165 infection group.

In our previous study, we have demonstrated that *H. parasuis* SH0165 infection activated NF-κB and p38/JNK MAPK signaling pathways to induce downstream inflammatory cytokines IL-8, CCL4, and CCL5 transcription (Chen et al., 2015a,b). Progress in elucidating mechanisms of crosstalk between autophagy and inflammatory signaling cascades have been studied (Cadwell, 2016). Thus, we next sought to determine whether the effect of autophagy on inflammation induced by SH0165 related with inflammatory signaling pathway. Firstly, we detected the effect of autophagy inducers and inhibitors on inflammatory signaling pathway during SH0165 infection. As shown in Figures 7A,B, rapamycin+SH0165 group reduced the activation of NF-κB, pp38, and pJNK compared with SH0165 group, while CQ enhanced the activation of NF-κB, pp38, and pJNK. However, 3MA+SH0165 enhanced the activation of pp38 but inhibit the expression of pp65 and pJNK compared with SH0165 group. Then siRNA of ATG were used. As shown in Figures 7C,D, siRNA knockdown of Beclin-1 and ATG7 enhanced the activation of NF-κB and pp38 by SH0165, while ATG5, Beclin-1 and ATG16L1 enhanced the activation of pJNK by SH0165 (Figures 7C,D). To further investigate the role of autophagy in SH0165-induced NF-κB activation, NF-κB luciferase reporter assay was performed. As shown in Figure 7E, rapamycin+SH0165 and 3MA+SH0165 group reduced the activation of NF-κB compared with SH0165 group, while CQ+SH0165 enhanced the activation of NF-κB. Since ATG can affect the secretion of cytokines, PK-15 cells were then transfected with pNF-κB-Luc and pRL-TK together with siRNAs of ATG5, Beclin-1, ATG7, ATG16L1, and NF-κB luciferase reporter assay was performed. Deficiency of Beclin-1 and ATG7 increased NF-κB activity in SH0165-infected cells (Figure 7F). In sum, these data suggested that autophagy inhibits SH0165-induced NF-κB, p38/JNK MAPK signaling pathway.

## Discussion

Virulence mechanisms are required for *H. parasuis* to establish infection and produce disease (Costa-Hurtado and Aragon, 2013). In literature there have been studies on comparing pathogenesis of virulent and non-virulent strains of *H. parasuis* (Zhang et al., 2016; Hua et al., 2018). It has been demonstrated that the highly virulent *H. parasuis* 5 (standard strain) induced a stronger autophagy level than less virulent *H. parasuis* 4 and avirulent *H. parasuis* 11 (Zhang et al., 2016). In our study, results from confocal microscopy, western blot and transmission electron microscopy (TEM) showed that highly virulent SH0165 (clinical strain) but not non-virulent HN0001 induced autophagy. We speculated that virulence factors of SH0165 may contribute to the bacterial pathogenesis, including adhesion to and invasion of host cells, resistance to phagocytosis by macrophages, induction of inflammation and also autophagy (Costa-Hurtado and Aragon, 2013; Chen et al., 2015a,b; Zhang et al., 2016). Comparisons of virulent and non-virulent strains of *H. parasuis* in functional assays have identified several virulence factors, such as hsdR, hsdS, gpT, and ompP2 (Yu et al., 2014). Further studies on virulence factors of SH0165 will provide a more complete understanding of the virulence mechanisms of *H. parasuis*.

Autophagosome accumulation is due to autophagy induction or a block in downstream steps of autophagy pathway, so it is necessary to analyze “autophagic fluxes” that distinguish between these two possibilities (Mizushima et al., 2010). As previously described, we traced autophagic fluxes with an mRFP-GFP-LC3 adenovirus (Hariharan et al., 2010, 2011). The fluorescent signal GFP quenches in the low PH inside the lysosome, and in contrast, fluorescence RFP are more stable in acidic compartments (Katayama et al., 2011). Autophagosomes and autolysosomes are labeled with yellow and red signals, respectively. Our results showed that both yellow and red puncta are increased in SH0165-infected cells, indicating autophagic flux is increased; however, 3-MA decreased both yellow and red puncta induced by SH0165 infection and autophagosome maturation is blocked. LC3 turnover assay is another method to measure autophagic flux (Mizushima et al., 2010). LC3-II formation has been detected in Figures 1C,D, 3E,F in our study. Different from western blot results of LC3 expression in Zhang's article (Zhang et al., 2016), we detected the transformation of LC3-I into its autophagosomal-associating form LC3-II. However, Zhang's article showed the expression of GFP-RFP-LC3 in cells infected with adenovirus expressing GFP-RFP-LC3. As shown in Figures 3E,F, levels of LC3-II in SH0165 plus reagents are decreased by treatment with 3-MA and increased by CQ compared with SH0165 infection only. Combining these results, CQ blocked the degradation of LC3-II and resulted in the accumulation of LC3-II, showing LC3 is delivered to lysosomes for degradation in the absence of lysosomal inhibitors in SH0165-infected cells.

Autophagy initiates with the formation of a phagophore, to which ATG proteins are recruited to form autophagosome (Virgin and Levine, 2009). Notably, most ATG gene deletions, knockdown or dominant-negative mutant block the formation of autophagosome. Cells lacking Atg5 (Mizushima et al., 2001), Becn1 (Qu et al., 2003), Atg7 (Komatsu et al., 2005), and Atg16l1 (Cadwell et al., 2008) have been confirmed to be autophagy deficiency. Our findings also demonstrated that knockdown of ATG5, Beclin-1, ATG7 and ATG16L1 inhibit autophagy induced by SH0165, indicating these ATG proteins are necessary for autophagosome generation in SH0165-infected cells.

In pathogenic bacteria, adhesion to and invasion of host cells contribute to crossing the cell barrier, surviving in the host, and penetration into deep tissues, ultimately leading to systemic disease (Vanier et al., 2006). Also in virulent *H. parasuis*, adhesion to and invasion of epithelial cells have been described as important steps of infection (Bouchet et al., 2009; Frandoloso et al., 2012). Previous studies have confirmed that *H. parasuis* can adhere to or invade PK-15 cells, porcine umbilicus vein endothelial cells (PUVEC), newborn pig tracheal cells (NPTr), and porcine alveolar macrophage cells (PAM) (Bouchet et al., 2009; Frandoloso et al., 2012; Zhang et al., 2012). Furthermore, it has been reported that pre-treat PK-15 cells with rapamycin inhibited the invasion of Hps5 standard strain (Zhang et al., 2016). This is consistent with what our finding that induction of autophagy inhibited the internalization of *H. parasuis* SH0165 in a dose-dependent manner. Moreover, our results go beyond previous reports, showing that loss of autophagy in PK-15 cells, either through knockdown of ATG5, Beclin-1, ATG7 or ATG16L1, or by treatment with the autophagy inhibitor CQ, increased the invasive number of *H. parasuis*. Unexpectedly, 3MA treatment decreased the number of invasive bacteria. In the previous studies, 3MA was reported to inhibit autophagy in *Brucella* and *Staphylococcus aureus* infection but significantly decreased the replication efficiency and the number of bacteria (Guo et al., 2012; Zhu et al., 2018). Similar to these bacteria, SH0165 may manipulate the autophagic machinery to their own benefit to a certain extent. Moreover, Wu et al. reported the dual role of 3MA in modulation of autophagy due to its differential temporal effects on class I and class III PI3K (Wu et al., 2010). We speculated that PI3K signaling inhibition affects cytoskeleton rearrangement necessary for bacterial entry (independently of autophagy). CQ may be a more suitable autophagy inhibitor in SH0165 treatment. However, the evolution of strategies to affect invasion of *H. parasuis* SH0165 by autophagy warrants more deep research.

Autophagy and inflammation are two interactive biological processes involved in bacterial infection. Recent studies showed rapamycin decreased the cytokines in AGS (a human gastric cancer cell line) cells infected with *Helicobacter pylori* (Li et al., 2017). Deficiency in autophagy proteins increases IL-1β levels which aggravates the degree of inflammation in a mouse model of gut inflammation (Saitoh et al., 2008). Consistent with these results, our results showed that the induction of autophagy with rapamycin inhibited the production of cytokines. Loss of autophagy proteins (ATG5, Beclin-1, ATG7, or ATG16L1) enhanced the secretion of cytokines IL-8, CCL4, and CCL5. Related inflammatory signaling pathways have been integrated into autophagy function that coordinate multicellular defense strategies (Deretic et al., 2013; Cadwell, 2016; Li et al., 2017). Our previous studies demonstrated that SH0165 infection induced inflammatory cytokines IL-8, CCL4, and CCL5 mediating by NF-κB and p38/JNK MAPK signaling pathways (Chen et al., 2012, 2015a,b). Therapeutically increased autophagy inhibited NF-κB signal pathway instead of MAPK pathway to limit the NLRP3 inflammasome and production of pro-inflammatory cytokine (Zhang et al., 2017). A similar pattern of results was obtained in this study, which indicated that the effect of autophagy on cytokine production during the SH0165 infection involved the NF-κB, pp38, and pJNK MAPK pathway, suggesting a new mechanism preventing inflammation.

In summary, the current study was carried out to explore mechanisms of autophagy induced by high-virulent serovar 5 strain *H. parasuis* SH0165. The role of autophagy in invasion and inflammation induced by SH0165 infection has been preliminarily studied. Factors involved in the *H. parasuis*-autophagy interplay warrant further research as potential preventive and therapy strategies for Glasser's disease.

## Author Contributions

CY, JL, WZ, and KH conducted the experiments. YW, GC, and DL analyzed the data. HJ and LX provided essential materials. YC and YZ designed and supervised the experiments and wrote and revised the manuscript.

### Conflict of Interest Statement

The authors declare that the research was conducted in the absence of any commercial or financial relationships that could be construed as a potential conflict of interest.
