# Supplementary material for: Autophagy Is a Defense Mechanism Inhibiting Invasion and Inflammation During High-Virulent Haemophilus parasuis Infection in PK-15 Cells
Source: Front Cell Infect Microbiol. 2019 Apr 16;9:93. doi: 10.3389/fcimb.2019.00093 (PMC6499186; doi:10.3389/fcimb.2019.00093)
Supplement: Supplementary file 1 [file Data_Sheet_1.pdf]

Fig.S1

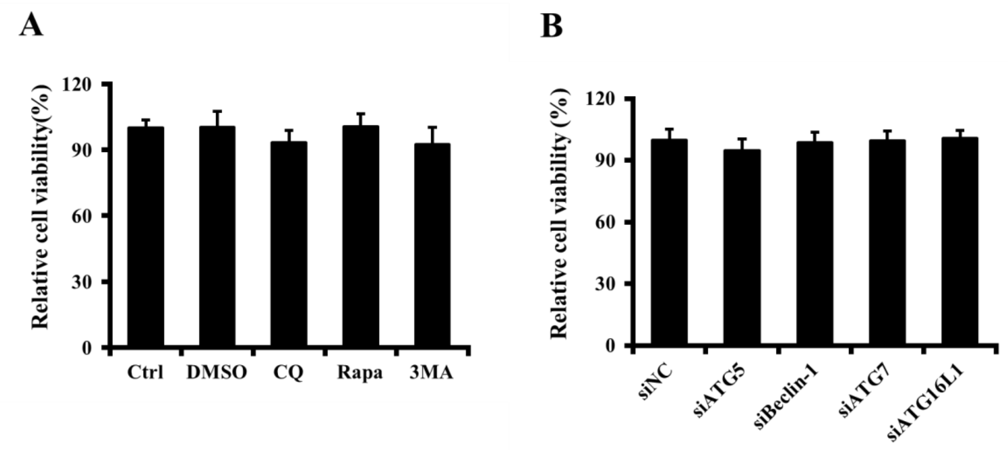

Fig.S1. Pharmacological or siRNA alterations of autophagy do not affect cell viability. Cell viability was determined by MTT assay after treatments with of 1 μM Rapa, 3 μM 3-MA or 10 μM CQ (**A**) and after transfection with siNegative, siATG5, siBeclin-1, siATG7 and siATG16L1 (**B**) for 36h. Percent relative cell viability is expressed as mean ± SD (n = 3).

Fig.S2

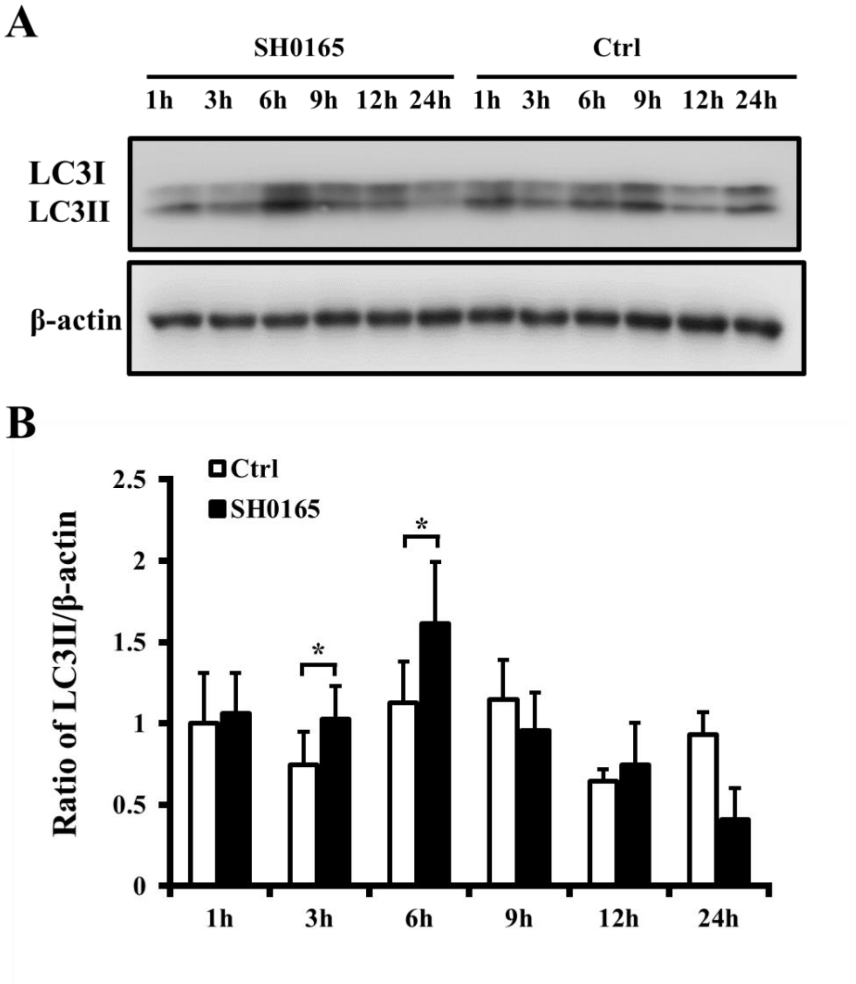

Fig. S2. Optimization of time of *H. parasuis* infection associated with autophagy.

(**A**) PK-15 cells were infected with *H. parasuis* SH0165 or uninfected (Ctrl) at 10^6CFU/mL for 1, 3, 6, 9, 12, 24h. (**B**) Analysis of LC3-II expression and its ratio to β-actin that was normalized to uninfection (Ctrl) set at 1.0. Data are reported as mean ± SD (n = 3; *$P < 0.05$ versus control group).

Fig.S3

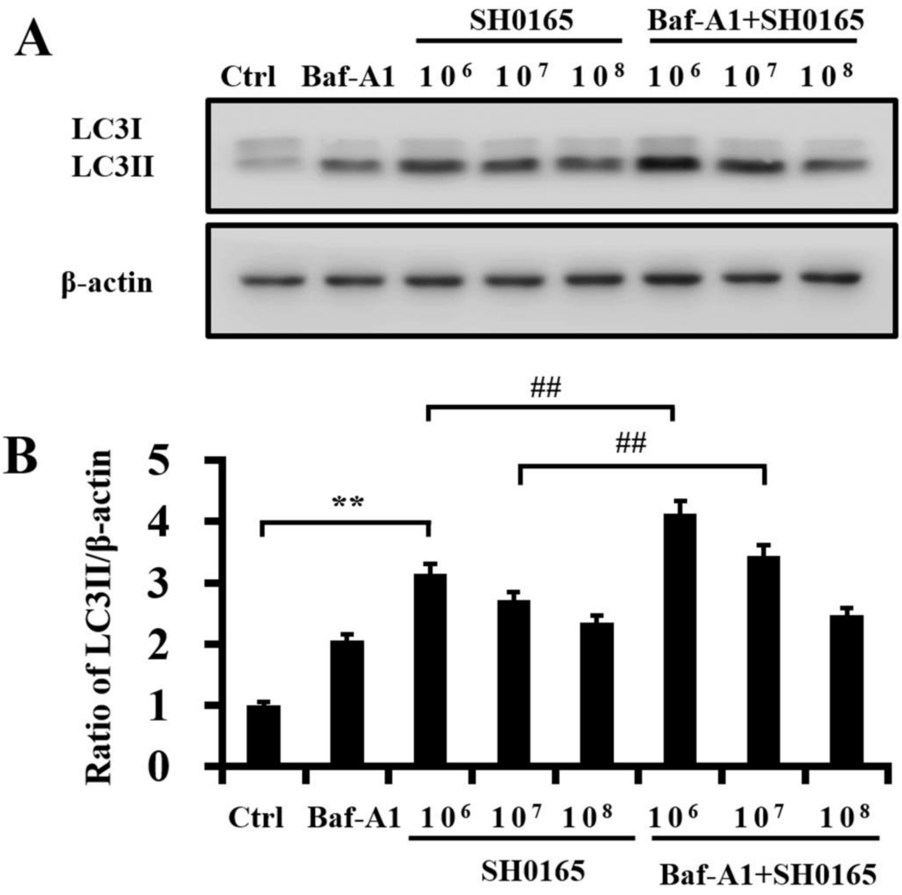

Fig. S3. LC3 protein were analyzed in absence and presence of Bafilomycin A1 in SH0165-infected cells. (**A**) Cells were treated with Baf-A1 (Baf-A1: 10 nM, 3 h) and then infected with SH0165 at 10^6, 10^7 or 10^8 CFU/mL for 6h. Cell lysates were analyzed for the expression of LC3-II by Western blot. (**B**) Densitometry was applied to quantify LC3 and β-actin protein density. The ratio of normalized LC3-II to β-actin; the data were presented as a mean ± SD from three independent experiments. $^{**}P <$ 0.01 versus control group. $^{\#\#}P < 0.01$ versus 10^6 and 10^7 group.

Fig.S4

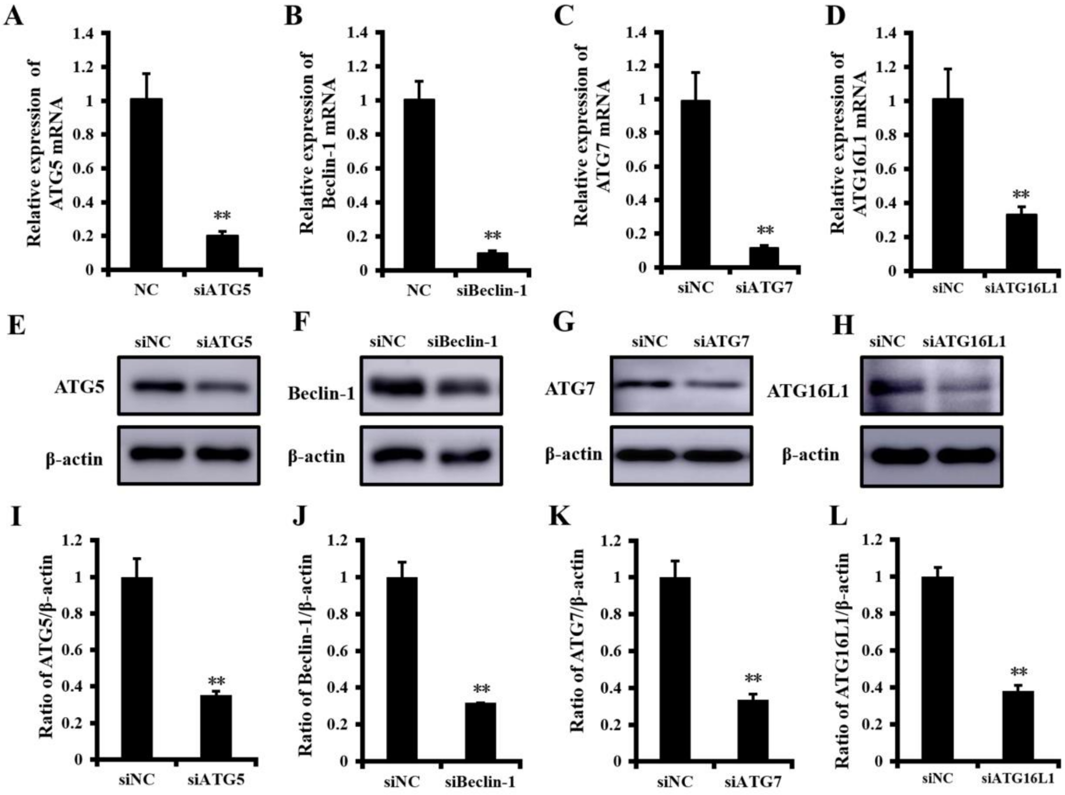

Fig.S4. Silencing efficiency of siRNA targeting siATG5, siBeclin-1, siATG7 and siATG16L1. PK-15 cells were transfected with siATG5, siBeclin-1, siATG7 and siATG16L1. After 24h, real time RT-PCR and Western blot were performed to analyze the expression of siATG5 [**(A)**, **(E)** and **(I)],** siBeclin-1 [**(B)**, **(F)** and **(J)],** siATG7 **[(C), (G)** and (**K**)] and siATG16L1 **[(D)**, **(H)** and **(L)]**. Data are reported as mean ± SD (n = 3; ***P* < 0.01 versus siNC group).

Fig.S5

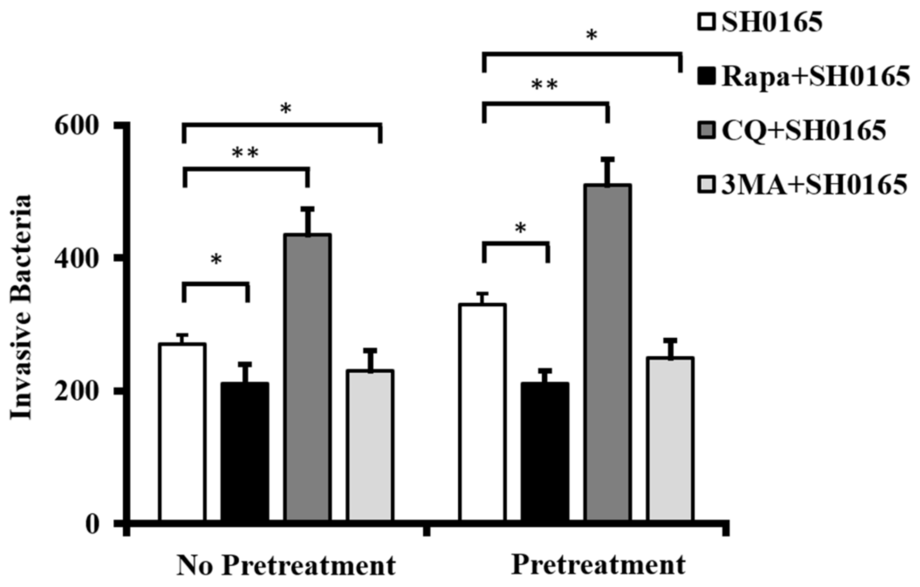

Fig.S5. Pretreatment of rapamycin, 3-MA or CQ do not affect the infection rate . PK-15 cells were pretreated or not pretreated with rapamycin (Rapa: 1 µM, 12 h), 3-MA (3mM, 3h) or chloroquine (CQ: 10 µM, 3h) followed by SH0165 infection at 10^7 CFU/mL. Invasion assay were performed as described in Materials and methods. The data shown as mean ± SD represent the number of bacteria that invaded the cells from three independent experiments performed in triplicate. *$P < 0.05$ and **$P < 0.05$as compared with SH0165 group.
